# Understanding how they *really* feel: Lesson learned from four approaches to soliciting user preferences for new contraceptive products in development

**DOI:** 10.12688/gatesopenres.14679.2

**Published:** 2024-01-08

**Authors:** Aurélie Brunie, Rebecca Callahan

**Affiliations:** 1 Health Services Research, FHI 360, Washington, District of Columbia, USA; 2 Product Development and Introduction, FHI 360, Durham, North Carolina, USA

**Keywords:** user preferences, acceptability, market research, contraception, family planning

## Abstract

**Background:**

An expanded range of contraceptive methods could reduce unintended pregnancies. User preferences research is important for successful development of products people want to use. This paper describes four approaches to preferences research soliciting user input in different ways: 1) perspectives on contraceptive method characteristics, 2) reactions to products in development, 3) trade-offs between contraceptive method characteristics, and 4) “blue-sky” ideas on novel contraceptive technologies.

**Methods:**

We conducted two mixed-method studies: one implemented in Burkina Faso and Uganda combining three of these approaches, and the other implemented in India and Nigeria using two approaches. We share observations on the strengths and weaknesses of each approach and draw on our experience to highlight lessons learned for future user preferences studies.

**Results:**

Each approach contributes to product development in different ways, and the usefulness of each methodology depends on the product development stage and corresponding informational needs.

**Conclusions:**

Recommendations for future research include combining different methods, angles, and perspectives; using sequential designs whenever possible; tailoring product descriptions to user understanding for optimal feedback; and acknowledging the value and limitations of both quantitative results for modeling demand and idiosyncratic ideas to inspire development of new products.

## Disclaimer

The views expressed in this article are those of the author(s). Publication in Gates Open Research does not imply endorsement by the Gates Foundation.

## Introduction

In low and middle income countries (LMICs), 218 million women want to avoid pregnancy but are not using modern contraceptives ^1^. In 2011, a study found that 70% of women with unmet needs in Sub-Saharan Africa, South Central Asia, and South-East Asia were not using modern contraception for reasons that could be addressed through adaptation to current methods or new contraceptive technologies ^2^. User preferences research focuses on the needs and desires of potential family planning users, eliciting data to inform contraceptive development so that new products will be adopted and used.

Despite the fact that contraceptives are widely used consumer products, surprisingly little consumer research has informed the development of new contraceptive technologies. Historically, technical feasibility has driven method design rather than the needs and preferences of eventual users. The dearth of user preferences research for new contraceptives is the result of multiple factors including the prominence of the provider in contraceptive decision-making and access, thus minimizing the importance of user preferences, and insufficient funding from public sector sources where a large portion of contraceptive product development has occurred ^3^. Notably, even the limited industry investment in contraceptive technology has not included meaningful user preferences research. While the World Health Organization (WHO) did recognize the importance of user input in the design of new technologies in its efforts as early as the 1970s when it created its Task Force on Acceptability Research in Family Planning ^4^, the vast majority of new contraceptive development over the past 40 years has suffered from insufficient input from its intended beneficiaries. 

Incorporating meaningful user preferences research into product research and development efforts is not without its challenges, however. Market research for technologies that do not yet exist involves measuring preferences for new products or product characteristics, which can provide insights into needs and preferences, but has unknown predictive validity for future behavior. Preferences are likely to vary depending on respondents’ life stage and prior reproductive and contraceptive experience, the social and cultural context, and a multitude of other personal, familial, and community factors. People’s perceptions and preferences change over time, as well, and particularly with increased exposure to new ideas and products. All of these factors affect the reliability and generalizability of user preference findings. While major “go/no-go” product development decisions should not be made solely on hypothetical findings, the data are important for identifying major gaps and problems, as well as opportunities for improving product design and desirability. 

At the time the research presented in this paper was conducted, several new contraceptive methods for women were under development or only available in limited markets, including a new, smaller copper intra-uterine device (Cu-IUD), a levonorgestrel-releasing intra-uterine device (hormonal IUD), a new single-rod implant, a biodegradable implant, a longer-acting injectable, a method of non-surgical permanent contraception, and a microarray patch platform for delivering contraceptive hormones (MAP). Work on these methods is partly informed by two recent user preferences research studies: one on the first six long-acting products that was conducted in Burkina Faso and Uganda (long-acting products or LAP study), and one on the MAP that was conducted in India and Nigeria (MAP study).

This paper outlines four different approaches we used to obtain user feedback, mostly from women but some with men, for the purposes of informing contraceptive product development across these two studies. The approaches solicited user input in different ways and broadly consisted of examining: 1) perspectives on contraceptive method characteristics, 2) reactions to the products in development, 3) trade-offs between contraceptive method characteristics, and 4) “blue-sky” ideas on novel contraceptive technologies. The aim of this paper is to highlight key considerations related to our experience implementing each of the four approaches and share lessons learned to inform the design and relevance of future contraceptive technology user preferences research.

## Methods

Designs for the two studies are described elsewhere ^5– 8^. Briefly, the LAP study used a cross-sectional, mixed-method design. In collaboration with the Performance Monitoring and Accountability 2020 (PMA2020) program, we added an acceptability module of 12 questions to the female questionnaire in Round 4 of PMA2020 surveys in Burkina Faso and Uganda. PMA2020 female surveys are conducted by female resident enumerators with all self-identifying women of reproductive age from a nationally representative sample of households ^9^. The acceptability module was completed by 2,743 women in Burkina Faso and 2,403 women in Uganda. It included questions on product characteristics and on the six long-acting products. In a separate qualitative component, we conducted 50 focus group discussions (FGDs) with self-identifying women, 10 FGDs with self-identifying men, 37 in-depth interviews (IDIs) with providers, and 15 key informant interviews across the two countries. Qualitative interviews covered experiences with actual contraceptives, a short ideation exercise, (that covered methods to be used by women, men, or both), and perspectives on the six products among all respondent groups. Women and providers also participated in a simple ranking exercise on important product characteristics.

The MAP study used a sequential, exploratory design. The first qualitative phase covered potential acceptability of the MAP for contraceptive delivery and perspectives on possible MAP characteristics through 16 FGDs and 20 IDIs with self-identifying women and 20 IDIs with providers across the two countries. Interviews included a proportional piling exercise using beads that allowed respondents to rank importance of MAP characteristics. A discrete choice experiment survey with 496 self-identifying women in India and 946 women in Nigeria followed the qualitative component.

Specific methods used for each of the four approaches are presented below, along with considerations for their design and implementation based on the authors’ experiences from the two studies. Table 1 matches data sources in the two studies with the four approaches. Table 2 synthesizes our view of the purpose and limitations of each approach. Throughout the rest of this paper, we differentiate between a product characteristic or *attribute* (e.g., duration of protection against pregnancy) and the different *levels* for this attribute (e.g., one month, three months, three years).

**Table 1.** Summary of data sources for each of the four approaches to soliciting user preferences.

	LAP study (Burkina Faso, Uganda)		MAP study (India, Nigeria)	
	PMA2020 module	Qualitative interviews	Qualitative interviews	DCE survey
**Perspectives ** **on method ** **characteristics**	Important characteristics in choosing a method ^**a**^ [W] Preferences for method duration ^**b**^ [W] Willingness to use a method causing amenorrhea ^**c**^ [W]	Simple ranking exercise on product characteristics [W,P]		
**Reactions to ** **the products in ** **development**	Interest in each of six new methods [W] Preferred method [W]	Perspectives on the six new methods [W, M, P, KI]	Perspectives on MAP [W, P]	
**Blue-sky ideas ** **on novel ** **contraceptive ** **technologies**		Blue-sky exercise [W, M, P]		
**Trade-offs ** **between ** **various method ** **characteristics**			Proportional piling exercise on MAP characteristics [W, P]	DCE [W]

Participant groups: W = Women; M = Men; P = Providers; KI = Key informants ^a^ “In choosing a method, what are the things about the method that are important to you?” ^b^ “If you could choose how often to take your contraceptive method, would you choose a method that you would take: every day, every time you have sex, every month or few months, every year or few years, once (it is permanent), other?” ^c^ “With some contraceptive methods, women do not get their period, but their period and fertility return when they stop using it. Would you choose a method that stops your period?”

**Table 2.** Purpose, illustrative measures and limitations of each approach.

Purpose	Illustrative measures	Limitations
**Perspectives on contraceptive method characteristics**		
• Prioritize method characteristics to target in product development	• Frequency distribution of preferred method characteristics	• Bound by list of characteristics anticipated by the researcher • No information on how to combine characteristics
**Reactions to product in development**		
• Prioritize methods for continued development efforts	• Overall interest in using the method • Perceived advantages and disadvantages of the method	• Complexity of concisely describing methods • Bound by method characteristics included in the description of the method • Social desirability bias
**Trade-offs between contraceptive method characteristics**		
• Inform potential adjustments to product design in terms of trade-offs between method characteristics	• Importance ranking of method characteristics	• Complexity of design balancing statistical considerations, cognitive burden on respondent, and technically feasible product designs • Bound by method characteristics included in the description of the method Unknown predictive validity
**Blue-sky ideas on novel contraceptive technologies**		
• Generate innovative ideas for new product designs	• List of user-generated ideas for potential novel product designs	• Difficulty abstracting from existing product designs among participants • Unknown external validity

## The four approaches

### Perspectives on method characteristics

***Description.*** This first approach gathers information on the characteristics that potential users want to see in new contraceptive products. This information can guide decisions on which attributes and/or levels to target in product development.

**Figure 1.**
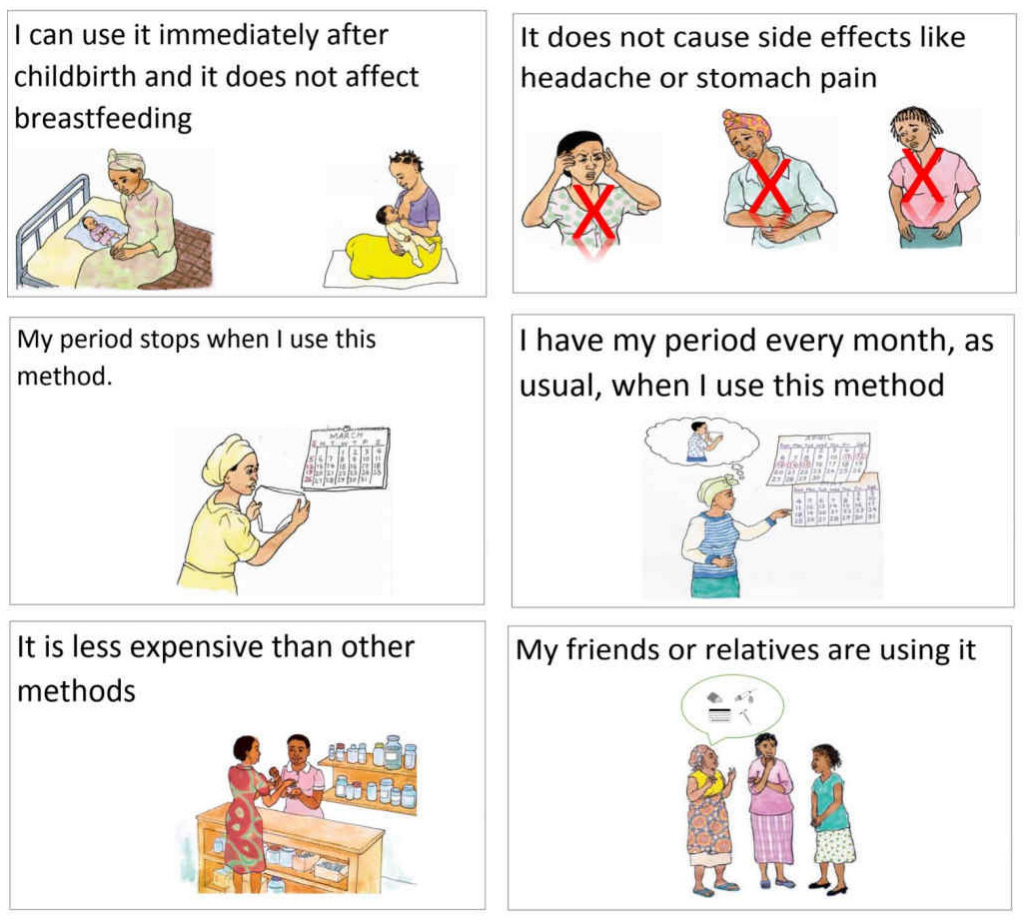
Examples of method characteristic cards that LAP (long-acting products) study participants were asked to rank in order of importance for method decision-making.

In the LAP study, we collected data on desired product characteristics from women through three questions in the PMA2020 survey acceptability module ^6^. The questions were framed broadly and not related to any contraceptive product. They included: important characteristics in choosing a method, preferences for method duration, and willingness to use a method causing amenorrhea. Qualitative interviews with women and providers also included a simple ranking exercise ^6^. Participants were asked to sort a set of cards with pre-written statements on product characteristics in order of importance to women in choosing a method ( Figure 1).

Data from the first survey question and the simple ranking exercise provided prioritized lists of important attributes, while survey questions on duration and amenorrhea offered insight into the acceptability of different attribute levels. As an example of the former, effectiveness, duration, and few side effects were the three attributes most commonly cited as important in choosing a method in both Burkina Faso and Uganda. Regarding attribute levels, with half or more women in Burkina Faso and Uganda saying they would like a method that lasts at least one year, the second survey question provides support for long-acting contraceptive products when it comes to duration of protection against pregnancy.

***Considerations for design and implementation.*** The survey and simple ranking provide different types of results and both suffer some limitations. With the survey question on important characteristics, responses are unprompted, and prioritization is based on the distribution of responses across participants. With the simple ranking exercise, participants react to a pre-specified list of items and prioritization is based on analysis of the ordering of the full list across interviews. Since everyone reacts to the same list, the ranking is more systematic, but it is also more limiting. In both cases, the value partly depends on the ability of the researcher to anticipate relevant characteristics, either as pre-coded survey response categories or as statements for the ranking, and on how they are formulated.

There is also a trade-off between the degree of specificity and the practicality of implementation (too many response categories or cards to rank), which both affect data quality. For example, survey results identified “duration” as important, whereas the statement “the method lasts for more than six months” ranked quite low on average in the simple ranking exercise. Yet “duration” is quite broad, and it is only through the second question on preferences for method duration that we acquired more details, with most women favoring methods they would take every year or every few years. These findings suggest that ranking results may have been different if we had used one year as a cut-off instead of six months. Including more detailed response categories or follow-up questions for all characteristics, however, may not always be possible, and some responses may therefore remain open to some degree of interpretation. In this study, this includes “effectiveness”, which may capture a range of responses from “protecting against pregnancy” to more precise specifications in terms of acceptable risk of method failure.

Additionally, while this approach of asking about preferred method characteristics tells us what attributes and/or levels users desire, it does not provide information on how to combine characteristics to achieve optimal demand. For example, although more women mentioned duration as important than few side effects, it does not necessarily mean that women will tolerate any kind of side effects if the method grants protection against pregnancy for a certain duration.

### Reactions to products in development

***Description.*** The second approach elicits reactions to specific contraceptive products by providing a description of the product and soliciting the perspectives of participants. It can inform priorities for continued product development by providing insight into which products may be most appealing to potential users, as well as into the desirability of the various characteristics of these products. We used this approach of soliciting reactions to product concepts with a range of methods at different stages of development. Some products like the hormonal IUD already exist, while others like the MAP are still more theoretical. The LAP study allowed some degree of comparison between the six methods, with a view to help prioritize development. The MAP study was focused on a single product, with the goal to inform design decisions.

**Figure 2.**
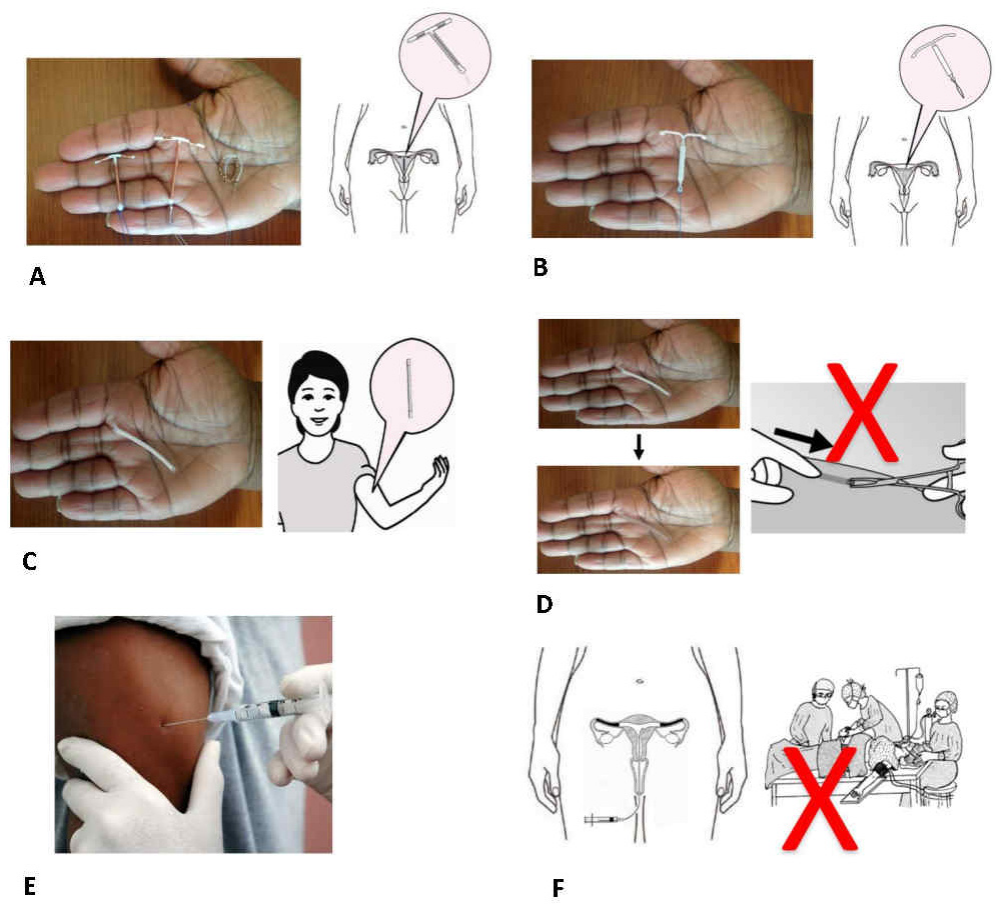
Examples of images used in the LAP (long-acting products) study to depict new methods in development or to be introduced. ( **A**) New copper IUD, ( **B**) Hormone-releasing IUD, ( **C**) Five-year single-rod implant, ( **D**) Biodegradable implant, ( **E**) Six-month injectable, ( **F**) Non-surgical permanent contraception.

In the LAP study, we described the six products in terms of their location, duration of effectiveness, potential for early removal or discontinuation, effect on menstruation, and whether they contained hormones ^5^. Descriptions included pictures ( Figure 2). In the PMA2020 acceptability module, we presented each of the six methods in turn and asked women if they would be interested in using each at any point in the future. We also asked respondents which method they preferred among all the products they said they were interested in, and, if applicable, their current method or a method they had used in the past 12 months. In the qualitative component with all participant groups, interviewers similarly introduced the six methods one-by-one with images and probed to understand perceived advantages and disadvantages.

In the MAP study, qualitative interviews solicited perspectives on advantages and disadvantages of the patch as a platform for contraceptive delivery ^7^. In addition, interviews introduced one-by-one a description of seven primary MAP attributes (option of self-application, pain at application, skin reaction, patch location, patch size, wear time, duration of effectiveness), soliciting participants’ perspectives on several possible levels for each characteristic (e.g., three possible patch sizes). We showed participants a close-up image of the potential product with microneedles and mock patches without needles that they could press onto their skin to mimic the experience with the actual product.

***Considerations for design and implementation.*** One advantage of the series of PMA2020 questions is that it can inform modeling of potential demand. Because there are other data on participant characteristics in the larger PMA2020 female questionnaire, it is also possible to cross-reference responses with participant characteristics. As an example, colleagues used data from the PMA2020 acceptability module to formulate assumptions on key inputs related to switching rates from other methods and uptake among women with unmet need for a demand forecast model for a six-month injectable (not publicly released). Qualitative interviews in both studies offer more details on what potential users like and do not like about products, which can provide guidance for subsequent development efforts or messaging.

One key challenge is the description of the methods. Some words, like “hormones,” do not easily translate into local languages, and other wordings such as “causing changes in the body” or “interfering with body processes” may not elicit the same reaction. A related challenge is the amount and format of the information being provided. In the LAP study, we used five characteristics to describe each method (location, duration, possibility of early removal, effect on period, and whether the method contained hormones). Particularly in the context of the PMA2020 acceptability module, it can be a lot of information for respondents to absorb, especially when switching rapidly from one method to the next. It is also critical to ensure that the delivery by data collectors is standardized so that all respondents react to the same information. Our strategy to ease the cognitive burden for the respondent while also streamlining interviewing was to structure method descriptions according to a small number of characteristics presented in a systematic way. Yet this approach risks leaving out other attributes that may be important to potential users, while conversely potentially focusing attention on attributes that respondents may otherwise not have considered. For example, in describing the LAI, we explained that side effects could not be reversed until the end of the six months as a counterpoint to saying that implants and IUDs could be removed at any time. In qualitative interviews, many participants emphasized lingering side effects as a disadvantage of the LAI; however, when they were discussing early removal of other methods, the focus tended to be on pregnancy intentions rather than side effects. The way methods are described and possible ensuing biases must be kept in mind when interpreting results.

Other limitations must be acknowledged that affect predictive validity. We cannot rule out some level of social desirability bias. Moreover, our estimates may not reflect demand for several reasons. First, we asked survey participants if they may be interested in using each product at any point in the future. In qualitative interviews, some participants who said they would consider using a product clearly qualified their response (e.g., when I no longer want children). The potentially time-dependent nature of demand may warrant additional attention for better projections. Second, demand is likely to be affected by other factors related to product introduction in real settings, such as provider capacity or pricing, that were not considered in the LAP study. Third, our goal in the LAP study was to help prioritize development efforts. Consequently, when we asked women about their preferred method in the survey, the choices were any of the six methods the respondent had expressed interest in and her current/recent method, as applicable. In real-world settings, demand for each method would be affected by the method mix, which would likely not include all six methods but would include other currently available products.

### Trade-offs between various method characteristics

***Description.*** The third approach of gauging user preferences generates information on the relative importance of product characteristics. It can inform potential adjustments to product design by guiding trade-offs between method characteristics.

**Figure 3.**
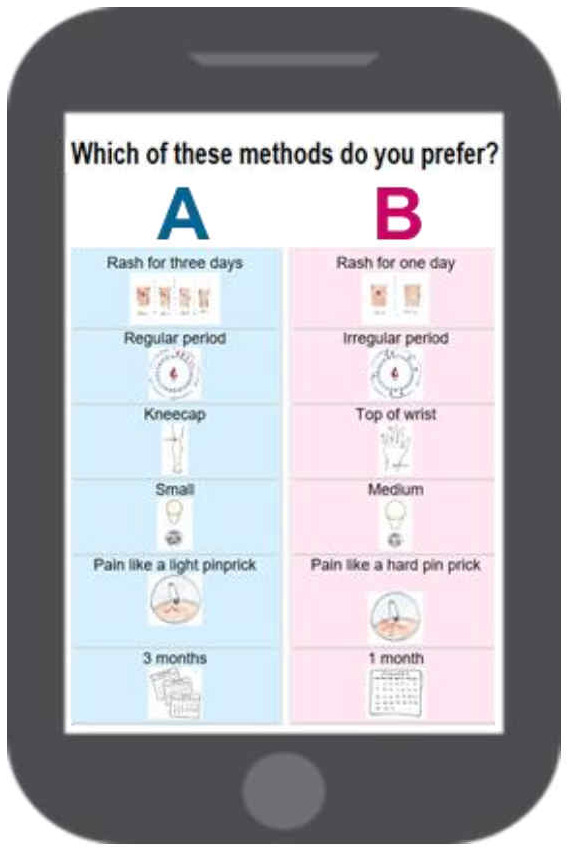
Example of a MAP (microarray patch platform) choice pair shown to respondents as part of the MAP DCE survey.

The qualitative component of the MAP study included a proportional piling exercise. We presented participants with a set of cards with pre-written statements on MAP characteristics, gave them a set number of beads and asked to distribute them across cards to reflect the relative importance of each statement to them in deciding whether to use the MAP. In the second phase of the study, we conducted a discrete choice experiment (DCE) to elicit potential users’ preferences for the MAP design ^8^. A DCE is a quantitative technique that involves asking respondents to state their preferred choice over alternative hypothetical scenarios. Here we presented women with 10 choice pairs of hypothetical MAPs described as bundles of six attributes (pain, skin reaction, location, size, duration, and effect on period) that vary in their levels (e.g., foot, kneecap, or wrist for location). Table 3 shows the attributes and their respective levels used in the DCE. Analysis of response data provides quantitative information on the strength of preference for each attribute level, as well as trade-offs. Prior to implementing the DCE, we conducted 10 cognitive interviews in India and eight in Nigeria to ensure proper implementation and test correct understanding of the exercise and method options by respondents. The survey was implemented on tablets, using both text and pictures ( Figure 3).

**Table 3.** Attributes and levels included in the MAP DCE survey.

Attribute	Levels
Effect on menstruation	• Regular period • Irregular period • Amenorrhea
Duration of effectiveness	• 1 month • 3 months • 6 months
Location of application	• Top of foot • Knee • Wrist
Pain	• Pain like hard pin prick • Pain like light pin prick • No pain
Rash at application site	• 1 day • 3 days
Patch size	• Small • Medium • Large (with reference to size of 1 rupee coin in India and bottle cap in Nigeria)

***Considerations for design and implementation.*** The proportional piling exercise is more sensitive than simple ranking because the bead count provides a measure of difference between items. However, it is a more limited expression of the trade-off approach than the DCE because only a few statements can be included, with each statement representing a specific value of one attribute level. In contrast, a DCE allows for permutation of attributes and levels to create many possible method descriptions. For example, our DCE had six attributes of two to three levels each, resulting in a total number of 486 possible combinations (method descriptions) and 235,710 possible pairs that can be shown to participants. The ability to elicit preferences for products that do not exist or for a much wider range of products than could actually be developed and made available is a well-recognized advantage of DCEs ^10^. The differentiating strength of this technique is that it allows the determination of how important each attribute is relative to others (relative importance by attribute), as well as understanding what specific levels within an attribute drive preferences (level part-worths). While these outputs may not be easily interpreted by all stakeholders, it is possible to run market simulations between two or more products to estimate the percentage of respondents who would prefer each and help prioritize specific combinations of attributes and levels for continued development.

While the rigor of DCEs is appealing, this approach is complex to design and implement. Preliminary qualitative work as was done in the MAP study is increasingly recommended in the DCE literature to inform attribute and level selection ^11– 14^. DCE design is guided by important decisions balancing statistical implications with the cognitive burden on the respondent ^15^. While DCE results allow for determination of combinations of attributes and levels for optimal acceptability, findings also need to be reconciled with what is technically feasible. Feasibility should already be considered when deriving the possible levels of each attribute; however, not all combinations of possible levels may be realistic. It is also important to note that findings on attribute importance and level values depend on the range of attribute and levels included in the DCE. For example, our DCE included three levels for the attribute pain at application: none, feeling like a light pinprick when the needles go in, and feeling like a hard/deep pinprick when the needles go in. Pain ranked fourth in order of importance in the analysis of results from India, with an importance value of 9%. With a wider range of levels including more acute pain, pain may have been more important. Similarly, an attribute importance is relative to the other attributes being included. In Nigeria, we implemented two versions of the DCE: one with all six attributes and one without effect on period but with the remaining five attributes. In the six-attribute version, the importance of duration relative to other attributes (including effect on period) was 25%; in the five-attribute version, it was 44%. 

Finally, the predictive validity of DCEs is not ascertained ^16^. Due to their hypothetical nature, DCEs are not constrained by what is actually currently available, contrary to other approaches such as randomized controlled experiments. The flip side of this is that DCEs reveal stated preferences among hypothetical choices, and these may not be the same as revealed preferences that may be observed if respondents were asked to make a real choice. 

### Blue-sky ideas on novel contraceptive technologies

***Description.*** The fourth approach captures users’ ideas about novel contraceptive products. It can help spur innovation in ways consistent with users’ preferences. In FGDs with women and with men in the LAP study, we asked participants to pair and brainstorm their ideal contraceptive method ^17, 18^. We instructed them to consider its delivery system, its duration of effectiveness, whether it would be used by women or men, and any other characteristics they thought were important. Each pair then reported on their method to the larger group, and the group had to agree on one method, which could be a method proposed by one of the pairs or a new idea spurred by the discussion. This exercise was also conducted individually with providers.

This approach to collecting user input follows a user-centered design process. It starts with potential users and engages them in a creative process to end up with contraceptive methods that are tailored to their needs. Because it places participants at the start of the creative process – rather than seek their reaction to methods already designed by scientists – it provides an opportunity to innovate in new directions. With this orientation, the blue-sky exercise we implemented borrows elements from human-centered design (HCD), a product development process long-used in the private sector that has become increasingly popular in global health in recent years. The full HCD process involves learning from potential users to understand their needs and circumstances, then ideating and conducting prototyping of possible solutions. Although earlier sections of the interviews helped with learning, the blue-sky exercise focused on creating ideas for new solutions in the context of a one-time interaction without any subsequent prototyping.

***Considerations for design and implementation.*** Although our intent was to identify potential novel designs, we found that many methods proposed by participants were based on common existing delivery mechanisms, namely injections, implants, and pills. Only 13% of proposed methods in Burkina Faso and 27% in Uganda were novel designs, such as creams, consumables, or wearables. In addition, over half of proposed methods were female-centered, and where methods were for use by men and women, there were several instances where it was clear from interview transcripts that participants added men as a potential target group after being queried by the moderator. One possible explanation for the high degree of similarity to existing methods may be that participants had difficulty abstracting from their actual experiences, particularly in the context of a short exercise within a larger interview that had already covered perspectives on and experiences with long-acting methods. One example supporting this explanation is that in Burkina Faso, a few participants justified proposing a particular delivery mechanism because they found the side effect profile of the corresponding current method tolerable. In addition, although they were instructed not to, moderators gave examples of existing delivery mechanisms to clarify instructions in a few FGDs. These limitations apply to both countries; however, it is worth noting that there was more innovation in Uganda than in Burkina Faso overall, possibly suggesting there may be cultural differences in terms of openness to thinking outside of the box. For example, although he eventually obliged and suggested his ideal method, a Burkinabe provider initially said that proposing an entirely new method was a job for scientists and “too much for him.”

The blue-sky exercise is structured to rapidly collect a range of user-generated ideas. When using this approach, other considerations must be addressed to sort and prioritize concepts for actual product development. User-generated ideas will need to be evaluated by scientists for the feasibility of their individual or combined attributes. Another inherent challenge is to build confidence that any idea may hold appeal beyond the participants who suggested it, including other participants who may have proposed other methods or those not directly involved in the research. After an initial assessment of feasibility, additional research may be needed to gather user input to justify continued development of the most promising feasible ideas. While the scope of the blue-sky exercise was constrained by the cross-sectional design and the other approaches being simultaneously implemented, others have recently implemented a more in-depth process in Kenya and Nigeria to ideate new contraceptive methods. This process included in-country market assessments engaging women, male partners, healthcare providers, and other stakeholders, followed by ideation events with a multidisciplinary group of local and global experts spanning product developers, tech start-ups, program managers, providers, and international procurement agencies ^19^.

## Lessons learned

**Investigate from a variety of angles and perspectives:** The two studies described here relied on triangulation to provide a comprehensive picture of user perspectives on and preferences for new contraceptive methods. We used triangulation on several levels, including of qualitative and quantitative methods, of study sites, of participant groups, and of the approaches described in this paper. Integrating qualitative and quantitative methods in the two studies allowed us to build confidence in findings while also adding depth of understanding. One particular strength of the LAP study was our collaboration with PMA2020, which permitted obtaining data from a nationally representative sample of women of reproductive age. The qualitative component expanded the inquiry to other important groups, including men, providers, and key informants, and captured more nuances on the context and circumstances behind participant perspectives. This added depth is important to understand not only what type of products may appeal to potential users, but also why to inform possible design changes, as well as introduction strategies through targeting and messaging. Incorporating several approaches in the design was also particularly helpful in corroborating findings. For example, interest in long-acting methods came out strongly across approaches, which helps address potential concerns over courtesy bias in soliciting reactions to the six new products.

**Consider opportunities for sequential or iterative designs to build on results:** In the MAP study, qualitative and quantitative methods were combined sequentially. The qualitative component supported the identification of relevant variables for the DCE survey, which was then administered to a much larger sample of women. At the same time, qualitative findings provided valuable information to interpret DCE results, for example illustrating why women preferred certain attribute levels to others, or some of the trade-offs they were making between attributes. The LAP study was cross-sectional but could have benefited from a sequential design to better inform the formulation of statements in the simple ranking exercise. A blue-sky approach could also benefit from an additional round of feedback on some of the ideated designs, and possibly from the incorporation of some prototyping. We believe that sequential or iterative designs should be considered whenever possible as they provide an opportunity to incorporate findings from one component into others. This can be particularly useful to provide more flexibility with user preferences research on new products when we do not have a good idea of what the research may find.

**Minimize potential biases in understanding of product descriptions:** Some of the approaches involve translating technical specifications for new products into appropriate and relevant user content to solicit feedback. One important consideration we noted in the two studies is the need to manage the cognitive burden that is placed on the respondent. In the LAP study, this mostly came from having to present six different products in the context of a single survey or qualitative interview. The MAP study covered only one product but with greater depth regarding attributes and several options regarding attribute levels. In both cases, there were clear trade-offs between the amount and specificity of information that could be included and the ease with which it could be comprehended. Both aspects warrant attention in designing study instruments and through additional pre-testing to ensure that all important points are conveyed clearly and also can be retained by participants. We found drawings to be particularly useful in the DCE to help participants keep track of the specific combination of attribute and levels presented to them for each choice pair. Adding visual aids can be a useful strategy both for clarification purposes and as a quick reference point. Cognitive interviews were also valuable to improve clarity and ensure comprehension of both wording and visual aids.

**Think twice when interpreting results for prioritization:** Numbers are seductive but when dealing with the possible adoption of new products in the future, modeling potential demand is particularly elusive. Predictive validity is a persisting challenge in hypothetical product acceptability studies. Just because people say they like a product does not mean they will use it. Our experience with the six products in the LAP study also serves as a reminder that interest in a product is likely to depend on the other options it is compared to. In the MAP study, preferences for MAP attributes are only valid within the limits of the attributes and levels specified in the design. Moreover, the duration of the product development cycle means that the people from whom feedback is solicited may no longer be potential users by the time products are available. Additionally, eventual demand will be influenced by the larger social, service delivery, and policy context in which products are introduced. To overcome these challenges, user preferences research should not be a one-time effort but should be fully integrated throughout the development cycle. At early stages, one-off, idiosyncratic ideas should also not be overlooked in qualitative forms of inquiry as they may gain momentum in subsequent rounds of research. Across approaches, we observed a tendency to err towards the familiar. Interest in the six products was highest for implants and injectables, and these delivery systems were also commonly suggested in the blue-sky exercise. Yet a moon-shot effort may be the needed game changer for product development to make a significant contribution towards overcoming long-standing barriers to contraceptive uptake and use.

## Conclusion

Our experience shows that there are similarities in the findings that are generated through different approaches to user preferences research. There are also clear differences and benefits to each approach and value in combining them to provide guidance related to prioritization as well as possible new development efforts. Keeping implementation and analysis manageable should also be an important consideration in choosing a particular approach or combination of approaches.

## Data Availability

No data are associated with this article.
