# Supplementary material for: Understanding how they really feel: Lesson learned from four approaches to soliciting user preferences for new contraceptive products in development
Source: Gates Open Res. 2024 Jan 8;7:81. Originally published 2023 May 30. [Version 2] doi: 10.12688/gatesopenres.14679.2 (PMC10914729; doi:10.12688/gatesopenres.14679.2)
Supplement: Peer review history

# Reviewer report: Reviewer response for version 2

**DOI:** 10.21956/gatesopenres.16602.r35761

**Published:** 2024-03-05

**Authors:** Barbara A. Friedland

**Affiliations:** 1 Center for Biomedical Research, Population Council, New York, NY, USA

The authors have successfully responded to my comments.

Does the article adequately reference differing views and opinions?

Yes

Are all factual statements correct, and are statements and arguments made adequately supported by citations?

Yes

Is the Open Letter written in accessible language?

Yes

Where applicable, are recommendations and next steps explained clearly for others to follow?

Yes

Is the rationale for the Open Letter provided in sufficient detail?

Yes

Reviewer Expertise:

HIV/contraceptive product development, end-user research to inform product development, stigma and other sociobehavioral research related to HIV/AIDS and unintended pregnancy

I confirm that I have read this submission and believe that I have an appropriate level of expertise to confirm that it is of an acceptable scientific standard.

# Reviewer report: Reviewer response for version 2

**DOI:** 10.21956/gatesopenres.16602.r35760

**Published:** 2024-02-29

**Authors:** Emily R Boniface

**Affiliations:** 1 Oregon Health & Science University, Portland, Oregon, USA

The revisions to the article, particularly the addition of Table 3, have improved the clarity. There are still a few very minor issues (see below), but otherwise all substantive concerns have been addressed.

1. I still believe that the title should read “Lessons learned” rather than “Lesson learned”
2. A bullet is still missing in the Limitations of the Trade-offs approach in Table 2.
3. LAI needs to be defined, or preferably fully written out since it’s a non-standard abbreviation and only used a few times, in the Reactions to products in development/Considerations section for, where the abbreviation appears twice.

Does the article adequately reference differing views and opinions?

Yes

Are all factual statements correct, and are statements and arguments made adequately supported by citations?

Yes

Is the Open Letter written in accessible language?

Partly

Where applicable, are recommendations and next steps explained clearly for others to follow?

Yes

Is the rationale for the Open Letter provided in sufficient detail?

Partly

Reviewer Expertise:

Family Planning, Public Health Policy

I confirm that I have read this submission and believe that I have an appropriate level of expertise to confirm that it is of an acceptable scientific standard.

# Reviewer report: Reviewer response for version 1

**DOI:** 10.21956/gatesopenres.16003.r35183

**Published:** 2023-11-21

**Authors:** Barbara A. Friedland

**Affiliations:** 1 Center for Biomedical Research, Population Council, New York, NY, USA

This is an excellent paper.  I have a few suggestions, as follows:

- I agree with the other reviewer that the description of the attributes and levels in DCE could be better explained. I have been involved in DCEs so I understand the concepts, but before I worked on one, it was complicated to understand.
- I would have liked to hear more about men's perspectives. The examples were from women and one provider, but it was not clear to me how the methods were working for men's input on contraceptives for women (or were they being asked about male contraception)?

Other than that, I have no additional comments.

Does the article adequately reference differing views and opinions?

Yes

Are all factual statements correct, and are statements and arguments made adequately supported by citations?

Yes

Is the Open Letter written in accessible language?

Yes

Where applicable, are recommendations and next steps explained clearly for others to follow?

Yes

Is the rationale for the Open Letter provided in sufficient detail?

Yes

Reviewer Expertise:

HIV/contraceptive product development, end-user research to inform product development, stigma and other sociobehavioral research related to HIV/AIDS and unintended pregnancy

I confirm that I have read this submission and believe that I have an appropriate level of expertise to confirm that it is of an acceptable scientific standard, however I have significant reservations, as outlined above.

# Response

**Published:** 2024-01-04

**Authors:** Rebecca Callahan

**Affiliations:** FHI360, USA

We added a table with attributes and levels in response to Reviewer #1.

 As shown in Table 1, men were included in the qualitative component of the LAP study where they were asked about their perspectives on the six new methods and participated in the blue skies activity.  As part of the latter exercise, men (and women) could come up with new ideas for male or female (or both) contraceptives.  We have added additional description to the Methods section on page 4 to clarify.

# Reviewer report: Reviewer response for version 1

**DOI:** 10.21956/gatesopenres.16003.r34578

**Published:** 2023-08-15

**Authors:** Emily R Boniface

**Affiliations:** 1 Oregon Health & Science University, Portland, Oregon, USA

This article synthesizes the work of two previous studies examining user preferences for new contraceptive methods in a LMIC setting. The article is well written and clearly outlines the lessons learned from both studies, including the value of using multiple methodologies and sequential designs to capture user preference data that are relevant and actionable. The article also thoughtfully articulates potential limitations of each approach, providing useful guidance for future studies of user preferences. However, minor changes could improve the framing and interpretability of the article.

**Rationale:**

- The introduction would benefit from a brief discussion of person-centeredness in the contraceptive domain. Although much of this work is focused on contraceptive counseling for existing methods (e.g., QCC, QCC-10, IQFP, and PCCC scales), the relationship between a focus on user needs and increased contraceptive uptake is relevant here.

**Language accessibility:**

- The title should read “Lessons learned” rather than “Lesson learned”
- The first sentence of the abstract should end with “use” rather than “us”
- The use of non-standard abbreviations for the various new methods feels unnecessary, and potentially confusing to the reader, since many are only used in Table 1. Suggest removing abbreviations for SRI, BDI, LAI, and NSPC.
- Suggest adding a brief explanation of proportional piling to the methods section where the term is initially used. Readers may be less familiar with this technique than the others mentioned, e.g., DCEs.
- The distinction between attribute and level is challenging to follow as presented, especially as the examples given for *level* change every time it is mentioned. Including a table that lists each attribute studied and their relevant levels would improve reader comprehension.
- A bullet is missing in the Limitations of the Trade-offs approach in Table 2.

Does the article adequately reference differing views and opinions?

Yes

Are all factual statements correct, and are statements and arguments made adequately supported by citations?

Yes

Is the Open Letter written in accessible language?

Partly

Where applicable, are recommendations and next steps explained clearly for others to follow?

Yes

Is the rationale for the Open Letter provided in sufficient detail?

Partly

Reviewer Expertise:

Family Planning, Public Health Policy

I confirm that I have read this submission and believe that I have an appropriate level of expertise to confirm that it is of an acceptable scientific standard, however I have significant reservations, as outlined above.

# Response

**Published:** 2024-01-04

**Authors:** Rebecca Callahan

**Affiliations:** FHI360, USA

**Rationale:**

- The introduction would benefit from a brief discussion of person-centeredness in the contraceptive domain. Although much of this work is focused on contraceptive counseling for existing methods (e.g., QCC, QCC-10, IQFP, and PCCC scales), the relationship between a focus on user needs and increased contraceptive uptake is relevant here. Thank you for this comment. *We agree that user preference research for new contraceptives is inherently person-centered.  We have revised the last sentence in the first paragraph of the Introduction to hopefully make the focus on the user clearer.  Given the length of the paper we have decided to not include a discussion about quality of counseling.*

**Language accessibility:**

- The title should read “Lessons learned” rather than “Lesson learned” *Corrected*.
- The first sentence of the abstract should end with “use” rather than “us” *Corrected.*
- The use of non-standard abbreviations for the various new methods feels unnecessary, and potentially confusing to the reader, since many are only used in Table 1. Suggest removing abbreviations for SRI, BDI, LAI, and NSPC. *Removed.*
- Suggest adding a brief explanation of proportional piling to the methods section where the term is initially used. Readers may be less familiar with this technique than the others mentioned, e.g., DCEs. *We added a brief description to page 4 where proportional piling is first mentioned.*
- The distinction between attribute and level is challenging to follow as presented, especially as the examples given for *level* change every time it is mentioned. Including a table that lists each attribute studied and their relevant levels would improve reader comprehension. *We added Table 3 showing the attributes and levels.*
- A bullet is missing in the Limitations of the Trade-offs approach in Table 2. *Corrected.*
